# Oxidation of Citalopram with Sodium Hypochlorite and Chlorine Dioxide: Influencing Factors and NDMA Formation Kinetics

**DOI:** 10.3390/molecules24173065

**Published:** 2019-08-23

**Authors:** Juan Lv, Yan Wang, Na Li

**Affiliations:** School of Environment and Architecture, University of Shanghai for Science and Technology, Shanghai 200093, China

**Keywords:** citalopram, *N*-nitrosodimethylamine, sodium hypochlorite, chlorine dioxide, oxidation, influencing factor, kinetics

## Abstract

The highly prescribed antidepressant, citalopram, as one of newly emerging pollutants, has been frequently detected in the aquatic environment. Citalopram oxidation was examined during sodium hypochlorite (NaOCl) and chlorine dioxide (ClO_2_) chlorination processes since conventional wastewater treatment plants cannot remove citalopram effectively. Citalopram has been demonstrated to form *N*-nitrosodimethylamine (NDMA) during chlorination in our previous study. Further investigation on NDMA formation kinetics was conducted in the present study. Influences of operational variables (disinfectant dose, pH value) and water matrix on citalopram degradation, as well as NDMA generation, were evaluated. The results indicated high reactivity of citalopram with NaOCl and ClO_2_. NDMA formation included two stages during CIT oxidation, which were linear related with reaction time. NaOCl was more beneficial to remove CIT, but it caused more NDMA formation. Increasing disinfectant dosage promoted citalopram removal and NDMA formation. However, no consistent correlation was found between citalopram removal and pH. Contrary to the situation of citalopram removal, NDMA generation was enhanced when citalopram was present in actual water matrices, especially in secondary effluent. DMA, as an intermediate of citalopram chlorination, contributed to NDMA formation, but not the only way.

## 1. Introduction

A higher prevalence of psychiatric disorders and increasing awareness of mental health issues caused the number of prescriptions for psychiatric pharmaceuticals, particularly antidepressants, promptly increasing [1]. Depression will be the world’s second most frequent illness by 2020 [2]. Selective serotonin reuptake inhibitors (SSRIs) antidepressants, which are also used to treat psychiatric disorders, panic disorder, and social phobia, have been widely marketed since the mid-1980s [3]. Recently, as a new class of emerging pollutants, antidepressants have caused worldwide concern due to their persistence and acute toxicity to aquatic life [4].

As a representative of SSRIs, citalopram (1-(3-dimethyaminopropyl-1-(4-fluoro-phenyl(-5- phthalancarbonitrile, CIT) is highly prescribed in USA [5], Northern Europe [6,7], and elsewhere in the world [8]. Between 12% and 23% of CIT is excreted unaltered in the urine due to the incomplete disintegration in human body [9]. Human excretion and disposal of unused or expired drugs in toilets may be the major input of CIT in the wastewater [10]. Wastewater treatment plants (WWTPs) cannot remove personal care products (PPCPs) residues and metabolites effectively since conventional WWTPs are not specifically designed to remove pharmaceutical and PPCPs. Styrishave B. et al. [11] revealed that abiotic degradation of CIT in the aquatic environment was low, and the removal of CIT was primarily due to sorption in the WWTPs. Lajeunesse et al. [12] reported that primary treatment and trickling filter/solids contact has limited capacity to remove antidepressants from sewage, and the removing efficiencies of CIT were between 3.5% and 48% from the study of five WWTPs in Canada. Besides that, the active metabolites and conjugates of psychoactive drugs could be transformed to their parent compounds during the treatment processes, thus causing the CIT concentration in effluent to be even higher than that in influent [13,14]. Consequently, WWTPs have been considered to be the major environment source of CIT to the surrounding aquatic environment. Several studies carried out in different countries reported the presence of CIT in different environmental matrices, including wastewater effluent (21–520 ng/L) [15], surface (2000 ng/L–8000 ng/L) [16], and ground water (40–90 ng/L) [17], in the order of ng/L [18] to µg/L.

The poor removal efficiency of CIT by conventional wastewater treatment has prompted some studies demonstrating that advanced oxidation processes (AOPs) were efficient at eliminating CIT, including O_3_, ClO_2_, UV, and Fenton oxidation [19,20]. It indicated that chemical oxidation could lead to CIT breakdown. Actually, ClO_2_ process is not only used as AOP, but also used as a powerful disinfectant in the WWTPs. Chlorination has been the most popular disinfection process for drinking water and wastewater because of strong oxidizing ability. Usually, disinfection is required as the last process step in WWTPs. CIT is still exposed to disinfection process due to the inefficient removal of CIT during the activated sludge process. However, there is limited information regarding the behavior of CIT during disinfection of wastewater.

Besides that, it cannot be ignored that one dimethylamine (DMA) group was contained in the structure of CIT (Table 1). It has been demonstrated that PPCPs containing DMA groups could be the potential precursors of *N*-nitrosodimethylamine (NDMA) with a high NDMA molecular conversion during chloramines and chlorination disinfection [21,22]. As a typical nitrosamine, NDMA is a suspected carcinogen with higher potency than trihalomethanes [23]. NDMA has been regulated at the ng/L level in drinking water in California, Ontario, and the European Union (EU) due to its frequent detection in the aquatic environment [24,25,26]. In our previous study [27], NDMA generation from seven psychoactive pharmaceuticals (including CIT) chlorination was revealed during sodium hypochlorite (NaOCl) and chlorine dioxide (ClO_2_) disinfection processes. It is important to understand the factors that may affect CIT degradation and NDMA formation in order to control the NDMA level after CIT chlorination in both drinking water and wastewater.

In this study, we investigated the characterizations of CIT oxidation during NaOCl and ClO_2_ chlorination processes. The kinetics of NDMA generation from CIT degradation was also clarified. Furthermore, the effects of disinfectant dosage, pH, and water matrix on CIT and NDMA formation were assessed in order to better control NDMA generation.

## 2. Results and Discussion

### 2.1. Effect of CIT Concentration

**Figure 1:**
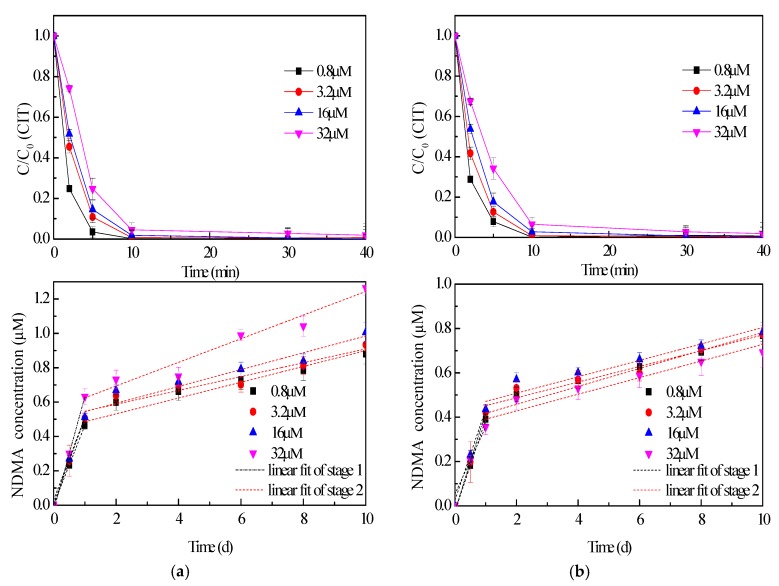
CIT removal and *N*-nitrosodimethylamine (NDMA) formation during CIT oxidation with NaOCl (**a**) and ClO_2_ (**b**) at different initial concentrations of CIT ([NaOCl] = [ClO_2_] = 1 mM, pH = 7.0) (Black dash lines represent linear fit of the first stage of NDMA generation, red dash lines represent linear fit of the second stage of NDMA generation).

As shown in Figure 1, the rapid elimination of CIT was observed once the chlorination process started, which was affected by the initial concentration of CIT. All of the CIT removal efficiencies exceeded 90% in 10 min., and then reached to 100% in 40 min. The control test results indicated the hydrolysis of CIT was negligible during the oxidation (Figure S1). It means that both NaOCl and ClO_2_ were effective on CIT degradation. Obviously, the removal of CIT was influenced by the initial concentration of CIT. It indicated that the disinfectant was insufficient to remove CIT rapidly when the initial amount of CIT was increased. Thus, it appeared that the degradation rate of CIT was decreased as the initial concentration increase. When comparing the C/C_0_ curves during the first 10 min. reaction when CIT treated by NaOCl with that treated by ClO_2_, it indicated that NaOCl was more beneficial in removing CIT, especially when the CIT concentration was higher (32 µM).

Furthermore, NDMA formation also proceeded during CIT oxidation. According to the results of NDMA concentration calculated using the equation (1) (Section 3.2), all of the NDMA molar yields were above 2%. More CIT being present in the solution caused more NDMA generation when CIT reacted with NaOCl. NDMA concentration after 10-day CIT oxidation with NaOCl increased from 0.88 μM to 1.26 μM as the CIT concentration increased from 0.8 μM to 32 μM. However, when 32 µM CIT was treated by ClO_2_, the amount of NDMA was lower than that under other conditions. It indicated that the dosage of the disinfectant limited the NDMA formation. Furthermore, the profiles of NDMA concentration presented two stages. NDMA was formed quickly during the first day of reaction (stage 1). More than half of NDMA amount appeared at the first stage of reaction. Afterwards, the NDMA concentration slowly increased during the rest of the 10-day oxidation (stage 2). Two kinetic models for NDMA formation were obtained by linear fits (Table S1), respectively, and all of the R^2^ values were above 0.9. For example, the generation rates of NDMA during NaOCl process went down from 5.35 × 10^−12^ M/s ~ 7.65 × 10^−12^ M/s to 5.32 × 10^−13^ M/s ~ 7.96 × 10^−13^ M/s when CIT was treated by NaOCl (Table S1). However, the profiles of NDMA concentration remained on an upward trend. Obviously, the contact time of CIT with disinfectant made a great influence on NDMA formation, especially at the stage 1. Since residual chlorine is persistent during drinking water delivery, it means that even a trace level of CIT presents in the drinking water might cause prolonged NDMA generation during the long distance water supply system. It is useful to pay attention to the risk of NDMA generation when CIT was distributed in raw water.

It is noteworthy that the CIT removal efficiencies reached nearly 100% just in 40 min. under the same test condition. The same phenomenon was also observed under other reaction conditions, including oxidation by ClO_2_ (Figure 1). In other words, NDMA generation still conducted, even if CIT was completely removed. It indicated that CIT decomposition did not directly result in NDMA formation. Some intermediate products from CIT decomposition also contributed to NDMA generation. It suggested that different NDMA precursors took part in the reaction, causing NDMA formation to appear in two stages. In addition, it seems that CIT treated by NaOCl tended to produce more NDMA than that treated by ClO_2_. It also indicated that ClO_2_ was a proper disinfectant for controlling NDMA formation.

### 2.2. Effect of Disinfectant Dose

**Figure 2:**
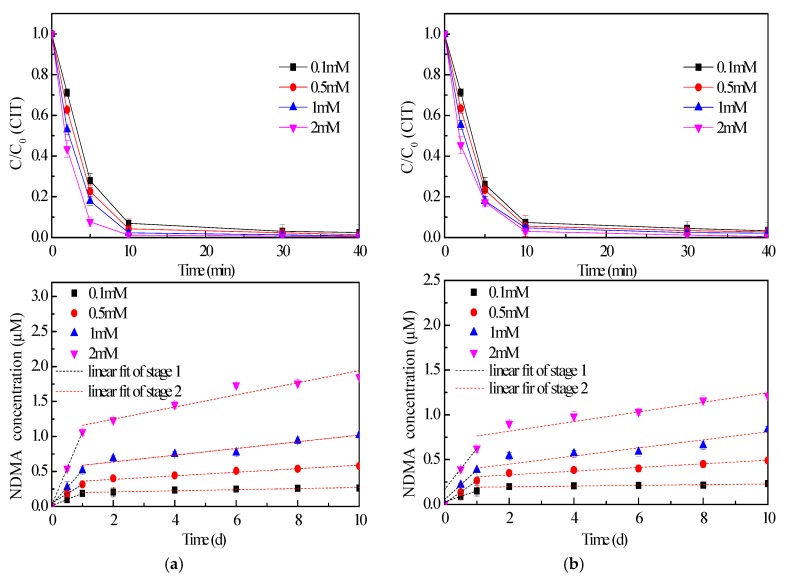
Effect of disinfectant dose on CIT removal and NDMA formation (NaOCl (**a**), ClO_2_ (**b**), C_0_ = 20 μM, pH = 7.0) (Black dash lines represent linear fit of the first stage of NDMA generation, red dash lines represent linear fit of the second stage of NDMA generation).

Effort was also made on the effect of disinfectant dose on CIT removal and NDMA formation kinetics since high reactivity of CIT during chlorination was identified (Figure 2). Apparently, increasing NaOCl concentration promoted CIT degradation. Especially at the first 5 min. of reaction, CIT removal efficiency was enhanced from 72.1% to 92.3% as the dosage of NaOCl increased from 0.1 mM to 2 mM. ClO_2_ concentration just made little improvement on CIT removal. Overall, CIT was completely removed in 40 min., even though the ClO_2_ dosage was only 0.1 mM.

However, increasing disinfectant dosages distinctly enhanced NDMA formation. The NDMA formation proceeded for 10 days in all cases, even if CIT was totally removed during 40 min. It also indicated that CIT decomposition caused NDMA generation through several steps. Two stages of NDMA generation were still observed, as Figure 2 shows. Moreover, increasing the disinfectant concentration enhanced NDMA generation from the intermediate products of CIT degradation, not only the production of NDMA, but also the rate of generation (Table S2). When the dosage of NaOCl increased from 0.1 mM to 2 mM, the rates of NDMA generation increased from 2.06 × 10^−12^ M/s to 1.20 × 10^−11^ M/s at stage 1 of NDMA generation (Table S2). Especially, there was little difference among the CIT removal efficiencies under given conditions, when CIT was treated by ClO_2_ (Figure 2b). However, the correlation of NDMA generation became poorer when the dosage of ClO_2_ decreased to 0.1 mM (Table S2). Furthermore, NDMA concentration at the end of 10-day reaction increased from 0.23 μM to 1.21 μM, owing to ClO_2_ concentration increased from 0.1 mM to 2 mM. It means that, although CIT could be removed efficiently, the transformation products could not be ignored, even when low dose disinfectant was supplied. The mixture of stable products, such as NDMA, might pose their own environmental and health risks.

### 2.3. Effect of pH Value on CIT Chlorination

**Figure 3:**
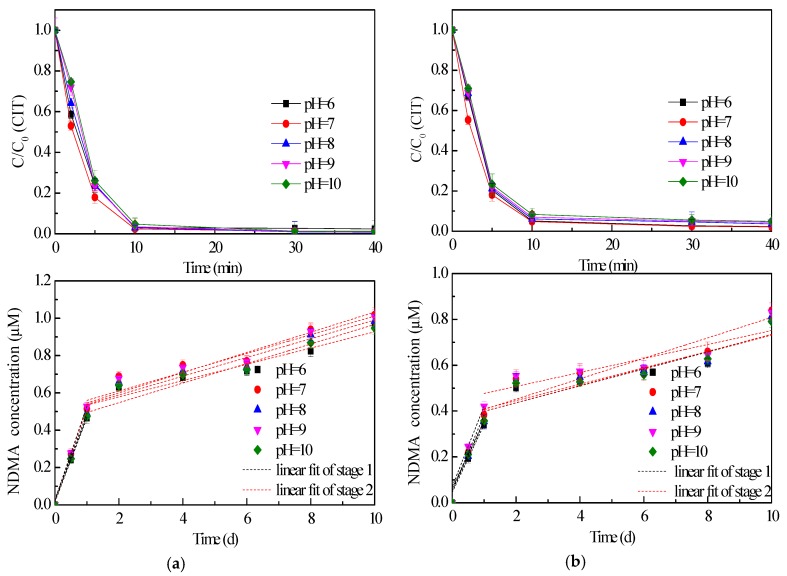
Effect of pH value on CIT removal and NDMA formation (NaOCl (**a**), ClO_2_ (**b**), C_0_ = 20 μM, [NaOCl] = [ClO_2_] = 1 mM) (Black dash lines represent linear fit of the first stage of NDMA generation, red dash lines represent linear fit of the second stage of NDMA generation).

A wide range of pH values (from 6 to 10) was tested to obtain deeper insight into the effect of pH on CIT removal and NDMA formation. All of the tests at different pH values showed the rapid oxidation of CIT during the first 10 min. of reaction (Figure 3). There was little difference in the curves of CIT removal efficiencies at different pH value. As described earlier, it seems that NaOCl was more beneficial for removing CIT. Consequently, more NDMA formation was observed during CIT oxidation with NaOCl. The pH value also had little influence on NDMA. Furthermore, there was just a little difference among the rates of NDMA formation both in stage 1 and stage 2 (Table S3). Therefore, it indicated that the pH value made little influence on both CIT composition and NDMA formation, especially in the pH range from 7 to 9.

It is well known that pH value influences the oxidation capacity and the speciation of NaOCl and ClO_2_. When CIT reacted with NaOCl at a different pH value, the degree of NaOCl dissociation (including OCl^−^ and HClO) depended on the pH value of the solution [28]. Since the pKa value of NaOCl is 7.5, it means that 50% of NaOCl is dissociated into OCl^−^ at pH 7.5. An increasing pH value can enhance the dissociation, thus reducing the oxidative capacity of NaOCl. In contrast, HClO was the dominant species under acidic conditions. The reaction between CIT and NaOCl could be promoted under acidic conditions, since HClO was more reactive than OCl^−^ [29]. According to previous literatures [30,31], ClO_2_ was more reactive under acidic conditions than neutral and alkaline conditions. Based on the above discussion, it indicated that the acidic condition was beneficial for enhancing the reactivity of disinfectant (NaOCl and ClO_2_).

Besides, the characteristics of CIT were also related to the pH value of solution. Since the pKa of CIT is 9.59 [8], it means that the fraction of deprotonated CIT increased in the pH range of 6–10. Furthermore, it has been demonstrated that both NaOCl and ClO_2_ react faster with deprotonated amines than protonated amines [32]. As typical tertiary amine, the reactions between CIT and disinfectant (NaOCl and ClO_2_) could be accelerated under alkaline condition. In conclusion, the effect of pH on CIT reactivity was contrary to that on the disinfectant oxidation capacity. Consequently, the effects of pH on disinfectant and CIT may cancel each other out. Thus, CIT removal seemed to be independent of pH value.

In the above discussion, two stages appeared during NDMA formation. It indicated that several intermediate products might react with NaOCl and ClO_2_ in steps, and then result in NDMA generation. The unidentified products and the reactions can also be affected by the pH value. Lots of effort was needed to explore the pathways of NDMA generation during CIT oxidation in the future.

### 2.4. Effect of Water Matrices on CIT Chlorination

**Figure 4:**
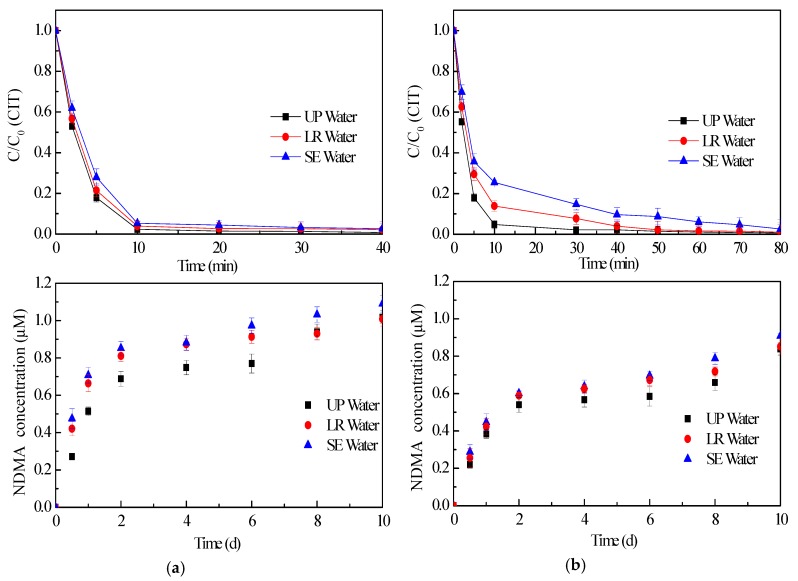
Effect of water matrices on CIT removal and NDMA formation (NaOCl (**a**), ClO_2_ (**b**), C_0_ = 20 μM, [NaOCl] = [ClO_2_] = 1 mM).

Under real treatment conditions, coexisting inorganic and organic compounds may affect the CIT oxidation. In other words, the effect of water matrices was also worthy of study. As the first step, experiments were performed while using surface water (taken from landscape river, LR Water) and secondary effluent (taken from wastewater treatment plant after wastewater treated by activated sludge, SE Water) in order to simulate real treatment conditions. Experiments were conducted in LR Water and SE Water with a spike of CIT since CIT was neither detected in LR Water nor in SE Water. Obviously, different water matrices caused various removal rates of CIT during oxidation. As shown in Figure 4, the CIT removal efficiencies in LR Water and SE Water were clearly lower than that in ultrapure water (UP Water) during the whole reaction. Coexisting inorganic and organic compounds in LR Water and SE Water were also oxidized since inorganic and organic micro pollutants can undergo reactions with NaOCl and ClO_2_ [32,33]. Thus, the reactions between CIT and disinfectant were impacted during the first 10 min. oxidation in both LR Water and SE Water. A lower degradation rate of CIT was observed in SE Water due to the higher TOC concentration in SE Water (16.2 mg/L) than that in LR Water (5.3 mg/L). As the reaction proceeded, the influence of water matrices became less. In addition, the final degradation efficiency of CIT was still nearly 100% when the oxidation lasted for 40 min. when CIT reacted with NaOCl. Obviously, it seems that the inhibition of water matrices was stronger when CIT was treated by ClO_2_. Complete removal of CIT achieved in 40 min. in UP Water. Nevertheless, the reaction time stretched to 80 min. when test was conducted in SE Water. It means that prolonging the chlorination time is beneficial in the CIT decomposition in the sewage treatment.

NDMA formation seems to be enhanced although CIT degradation was impressed both in LR Water and SE Water. Take characterizations of SE Water into consideration, it seems that not only CIT contributed to NDMA formation, coexistent compounds were also involved in NDMA generation. The compounds in surface water (LR Water) and secondary effluent (SE Water) included inorganic matter, natural organic matter (NOM), and synthetic organic matter, which could influence NDMA formation in different ways. Bromide was reported to either catalyze [34,35] or inhibit [36] NDMA formation. However, the bromide concentrations in LR Water (8 μg/L) and SE water (12 μg/L) were much lower than that in those studies. Thus, the effect of bromide during CIT oxidation was negligible. Besides bromide, NH_4_^+^ was detected in both LR Water and SE Water (Table 2). NH_4_^+^ could react with NaOCl and then cause chloramine formation. Chloramination was more propitious to NDMA generation when compared with NaOCl and ClO_2_ processes [37,38]. Thus, the presence of NH_4_^+^ in actual wastewater might enhance NDMA formation during CIT treated by NaOCl.

NOM could affect CIT removal and NDMA formation in several ways. Firstly, the competition for oxidant from NOM was limited, due to the large excess of disinfectant relative to the CIT (1 mM vs. 20 μM) during the oxidation. Secondly, since the pKa of CIT was 9.59, the possible electrostatic attraction between positive CIT and negatively charged NOM may occur in natural water. De Ridder et al. [39] also found the electrostatic attraction between negatively charged NOM and positively charged pharmaceuticals. Furthermore, the electrostatic attraction may hinder the decomposition of CIT, and thereby suppress NDMA formation through obstructing CIT decomposition. However, no reduction of NDMA generation was observed during the tests, although CIT removal was depressed. It also indicated that the influence of electrostatic attraction from NOM on NDMA formation was considered to be minimal. Besides these discussed above, it cannot be ignored that NOM has been revealed as the precursor of NDMA during water chloramination and chlorination [40]. The NOM in LR Water and SE Water likely produced NDMA during chlorination. Thus, tests of NDMA generation from LR Water and SE Water without CIT spiking were also conducted under the same test conditions. As Figure S2 shows, LR Water and SE Water chlorination indeed led to NDMA generation without CIT addition. Obviously, SE Water caused more NDMA formation, especially when reacting with NaOCl. The NDMA concentration from SE Water was lower than 500 ng/L. However, the differences of NDMA concentration between tested in SE Water and UP Water were nearly 5.3 μg/L (0.072 μM), both in NaOCl process and ClO_2_ process (Figure 4). It means that the NDMA formation from the background water could not account for NDMA yields increase. Furthermore, it has been verified that tertiary amines can result in chlorammonium species (R_3_N^+^-Cl) generation during aqueous chlorine disinfection [41]. R_3_N^+^-Cl can potentially enhance organic contaminants transformation rate up to three orders of magnitude and then largely accelerate disinfection by-product formation. However, the reactions of organic contaminants with R_3_N^+^-Cl did generate similar by-products to those from aqueous chlorine. This indicates that R_3_N^+^-Cl formation during CIT chlorination may increase the rate of NOM oxidation and then enhance NDMA generation. Further research is necessary to confirm the effect of NOM in the actual water matrices on NDMA formation during chlorination.

### 2.5. Possible NDMA Formation Mechanisms

**Figure 5:**
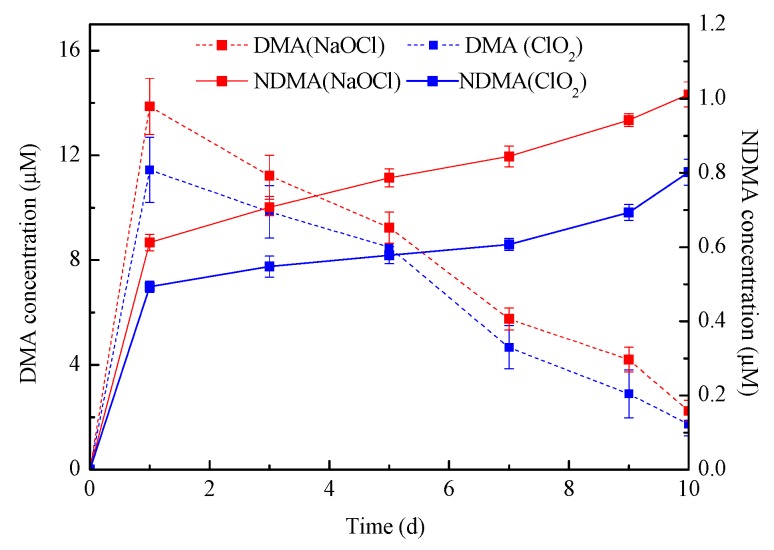
Dimethylamine (DMA) and NDMA formation during CIT oxidation with NaOCl and ClO_2_ (C_0_ = 20µM, [NaOCl] = [ClO_2_] = 1 mM, pH = 7).

As a member of tertiary alkylamines, the DMA group in CIT structure indicated that CIT could be a potential precursor of NDMA during chlorination and chloramination. Mitch and Schreiber [42] demonstrated that tertiary alkylamines were rapidly degraded during chlorination to form secondary alkylamines and aldehydes quantitatively (Scheme S1). The oxidation of tertiary amines with ClO_2_ also caused DMA generation via the dealkylation process initiated by a one-electron oxidation [43] (Scheme S2). The further oxidation of secondary alkylamines, such as DMA, caused NDMA formation. Furthermore, NDMA generation during the reaction of DMA with NaOCl or ClO_2_ has been confirmed in several studies [37,38,44]. Thus, effort was also made to investigate the DMA generation during CIT chlorination (Figure 5). Obviously, DMA formation was observed as reaction proceeding. DMA concentration was up to 13.9 μM after CIT chlorination for one day, and then declined to 2.24 μM at the end of reaction. The removal efficiency of CIT was near 100% just in 40 min. when compared the results in Section 2.1 (Figure 1). It also indicated that DMA was not produced from CIT decomposition directly. The first step of the reaction is an electrophilic chlorine substitution (Scheme S1), the nature of the moieties close to the DMA group can influence the reaction and affect DMA formation. Generally, an electron-withdrawing group (EWG) can decrease the electron density and hinder the chlorine transfer. Conversely, an electron-donating group (EDG) that is close to the DMA group increases the electron density on the nitrogen atom and help in chlorine substitution. Moreover, the steric hindrance between the EDG/EWG and the electrophile (chlorine) also influenced the reactivity. Obviously, the DMA group was not directly connected the EDG in CIT, which weakened the electron-donating effects.

Besides that, the rapid generation of DMA and NDMA both appeared during the first 24 h (stage 1) of reaction. Moreover, the peak value of DMA reached 13.9 μM in NaOCl chlorination, which was lower than the theoretical value of DMA formation (20 μM) in a 1:1 molar ratio of CIT. Subsequently, DMA concentration sharply declined with proceeding chlorination. At the same time, the NDMA concentration continuously increased (stage 1). However, the rate of NDMA formation in stage 2 was much slower than that in stage 1 (Table S2). DMA decline meant the precursor of NDMA reducing. Consequently, NDMA generation was gradually weakened due to DMA loss. Thus, the variations of DMA concentration were consistent with the two stages of NDMA formation. It also confirmed that DMA oxidation contributed to NDMA generation. However, the pathway of NDMA formation from DMA chlorination has not been identified [37,45]. Especially, the mechanism of nitroso-group formation in the NDMA is still unclear so far. It was assumed that nitrite was formed during the oxidation of DMA by chlorination [45]. Thus, the mechanism of NDMA formation during DMA chlorination is worthy of further study.

It is noteworthy that more DMA generation was observed when NaOCl treated CIT. Chang et al. [38] also suggested that the NDMA formation potentials of DMA during chlorination followed the order of NaOCl > ClO_2_. As mentioned above, CIT oxidation caused more NDMA formation during CIT reacted with NaOCl. It also identified that the DMA produced during CIT oxidation caused NDMA formation. Based on the above discussion, it stated that more emphasis should be placed on reducing DMA formation from CIT degradation. Besides, according to the results of previous studies, all of the NDMA molar yields from DMA chlorination were below 1% [38,45]. It was lower than the molar yield from CIT oxidation (higher than 3%). It suggested that DMA was involved in NDMA generation, but it was not the only way for NDMA formation. There was another possible pathway for the NDMA formation from CIT not involving DMA production.

## 3. Materials and Methods

### 3.1. Chemicals

CIT, NDMA-d6, and NDMA were purchased from Chem Service Inc. (West Chester, PA, USA). Methanol and acetonitrile were obtained from Sigma Chemical Co. (St. Louis, MO, USA). UP water was prepared with a Gradient A10 water purification system (Millipore, Bedford, MA, USA). The other reagents that were used in this study were of analytical grade and supplied by Sinopharm Chemical Reagent Co. Ltd. (Shanghai, China).

NaOCl solution was freshly diluted from NaOCl stock solutions. ClO_2_ solution was freshly prepared by dilution of ClO_2_ stock solution prepared by the reaction of the two powder reagents from Twin Oxide (De Tongelreep, Netherlands). All of the disinfectant solutions concentrations were determined before disinfection processes.

### 3.2. Chlorination Experiments Procedure

The experiments were conducted at 20 °C in sealed 1L brown amber glass bottles after disinfectant (NaOCl or ClO_2_) addition, and the solution volume of each bottle was 900 mL. The bottles were placed in a temperature-controlled shaker for 10 days. All of the experiments were performed in triplicate. Glassware used in this study was rinsed with acetone and baked at 400 °C for 6 h prior to use. Except where otherwise stated, all the water solutions were prepared by buffer solution (phosphate buffer) at pH 7.0. During the course of experiments, the samples were collected at predefined time points and filtered through 0.45 μm nylon filers for analysis. All reactions were quenched by addition of excess ascorbic acid solution (10 mM) to quench the chlorinating agent.

The initial concentration of CIT was set at from 0.8 µM to 32 µM to investigate CIT oxidation during chlorination in order to identify NDMA formation. Solution containing CIT was exposed to 1mM of disinfectant (NaOCl or ClO_2_) for 10 days. The impact of disinfectant dosage was tested at four doses (0.1 mM, 0.5 mM, 1 mM, and 2 mM). For the evaluation of pH value influence, pH was adjusted from 5 to 8 with phosphate buffer, and adjusted from 9 to 10 with borate buffer and borax buffer. CIT was frequently detected in natural water and wastewater. Chlorination of CIT in LR Water and SE Water was also conducted under the same test conditions. The LR Water and SE Water samples were obtained from the landscape river located in University of Shanghai for science and technology, and the secondary wastewater effluent from Quyang wastewater treatment plant in Shanghai, respectively. Table 2 lists the characteristics of LR Water and SE Water. All water samples were filtered (0.45 μm cellulose nitrate) within 24 h after sampling and then stored at 4 °C prior to the addition of chlorine and CIT.

Molar yields of NDMA from CIT chlorination were calculated while using the equation below.

(1) $$Y_{\mathrm{NDMA}-m}(\%)=\frac{{\left[\mathrm{NDMA}\right]}_{m}}{{\left[M\right]}_{0}}\times 100\%$$

where [NDMA]_m_ (mM) is the NDMA concentration that formed after disinfection and [M]_0_(mM) represents the initial concentration of CIT.

### 3.3. Analytical Methods

CIT concentration was determined by ultra-performance liquid chromatography (Thermo Fisher Scientific, Waltham, MA, USA), equipped with an auto sampler connected to a mass spectrometer (UPLC-ESI/MS, Thermo). An Eclipse XDB C18 column (150 × 2.1 mm, 3.5 mm; Agilent, Foster City, CA, USA) was used for separation. The mobile phase was composed of acetonitrile and ultrapure water (*v*/*v* = 60:40) with the injection volume of 10 µL and the flow rate of 100 µL/min. at 30 °C. Positive electro spray ionization combined with selective reaction monitoring. The optimal ion spray parameters were as follows: auxiliary gas (N_2_) at 34.5 kPa, collision gas (Ar) at 0.16 kPa, and ion spray voltage at 3800V. The SRM transitions were m/z 325-109 for qualitative analysis. The detection limit of the instrument was 10 μg/L.

The samples were analyzed for NDMA concentration while using solid-phase extraction (SPE, Restek cat.#26032, Restek, Bellefonte, PA, USA) followed by UPLC-ESI/MS [46]. The UPLC-ESI/MS methods have been described in previously study [47]. The method detection limit (MDL) for NDMA was 4 ng/L.

## 4. Conclusions

NaOCl and ClO_2_ were both effective in CIT removal. CIT oxidation resulted in NDMA and DMA generation. NDMA formation during CIT oxidation included two stages, both having linear relationship with reaction time. CIT removal and NDMA formation were independent of the pH value. Higher dosage of disinfectant accelerated CIT removal. More NDMA was generated when CIT oxidaition was conducted under actual water matrices, especially in SE Water. DMA formed from CIT degradation contributed to NDMA generation. There might be other possible pathways for NDMA formation not involving DMA. In addition, NaOCl was more beneficial for removing CIT, but caused more NDMA formation. ClO_2_ is expected to be a proper disinfectant to reduce NDMA generation risk from CIT during chlorination process.

## Figures and Tables

**Table 1:** Chemical structure and pKa [8] of citalopram (1-(3-dimethyaminopropyl-1-(4-fluoro-phenyl(-5- phthalancarbonitrile (CIT)).

Compound Investigated	Molecular Structure	pKa	CAS
Citalopram	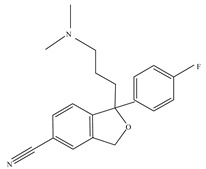	9.59	59729-33-8

**Table 2:** Characteristics of LR Water and SE Water.

Water Samples	pH	HCO_3_^−^ (mg/L)	TOC (mg/L)	TN (mg/L)	NH_4_^+^-N (mg/L)
LR Water	7.18	85.3	5.3	2.38	0.07
SE Water	6.88	284.3	16.2	13.25	0.18

## Supplementary Materials

The following are available online, Figure S1: Control test of CIT hydrolysis (C_0_ = 20 μM, pH = 7.0), Figure S2: NDMA formation during chlorination of LR Water and SE Water without drugs addition ([NaOCl] = [ClO_2_] = 1mM), Table S1: Kinetics of NDMA generation during CIT oxidation with NaOCl and ClO_2_ at different initial CIT concentrations, Table S2: Kinetics of NDMA generation during CIT oxidation with NaOCl and ClO_2_ at different disinfectant doses, Table S3: Kinetics of NDMA generation during CIT oxidation with NaOCl and ClO_2_ at different pH values, Scheme S1: Mechanism of tertiary alkylamines degradation during chlorination [42], Scheme S2: Mechanism of the reaction of tertiary amines containing NDMA-precursors with ClO_2_ [43].
