# Supplementary material for: Oxidation of Citalopram with Sodium Hypochlorite and Chlorine Dioxide: Influencing Factors and NDMA Formation Kinetics
Source: Molecules. 2019 Aug 23;24(17):3065. doi: 10.3390/molecules24173065 (PMC6749231; doi:10.3390/molecules24173065)
Supplement: Supplementary file 1 [file molecules-24-03065-s001.pdf]

Oxidation of Citalopram with Sodium Hypochlorite and Chlorine Dioxide: Influencing Factors and NDMA Formation Kinetics

Juan Lv *, Yan Wang and Na Li

School of Environment and Architecture, University of Shanghai for Science and Technology, Shanghai 200093, China; lujuan@usst.edu.cn; Tel.: +86-21-5527-5979

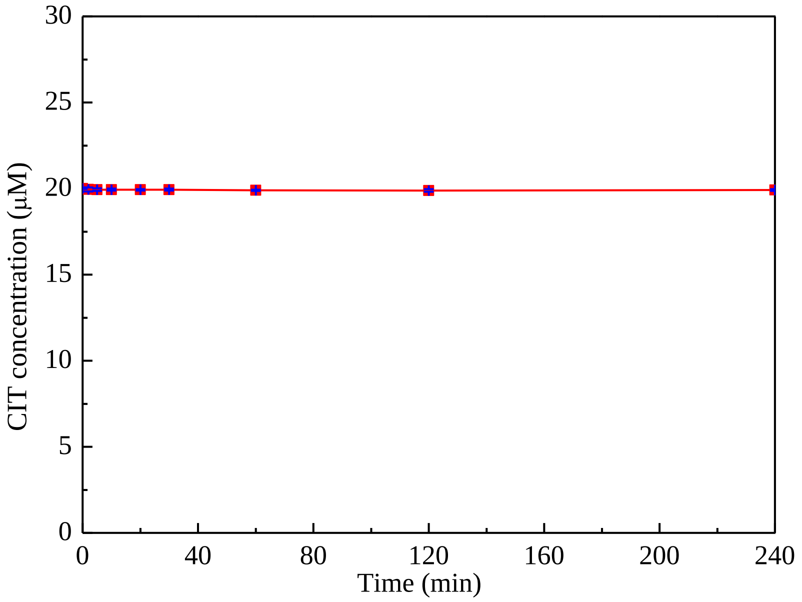

Figure S1. Control test of CIT hydrolysis (C_0= 20 μM, pH = 7.0).

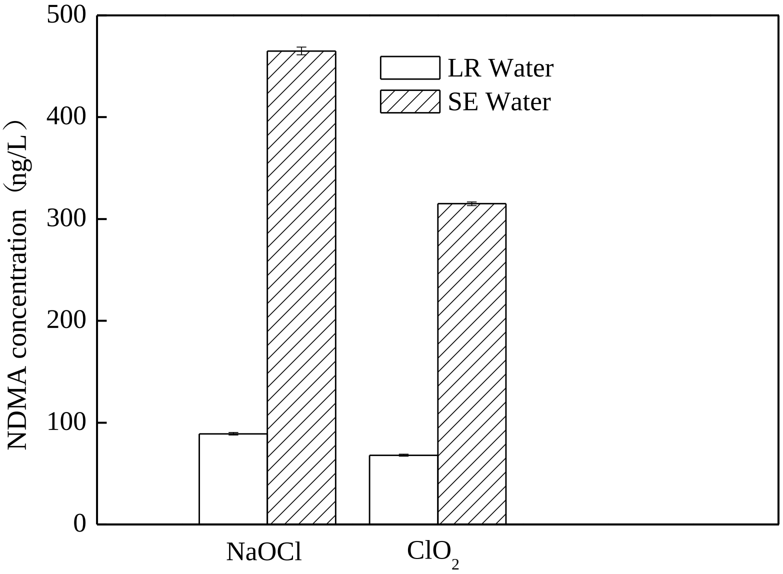

Figure S2. NDMA formation during chlorination of LR Water and SE Water without CIT addition ([NaOCl] = [ClO_2] = 1mM).

Table 1. Kinetics of NDMA generation during CIT oxidation with NaOCl and ClO_2 at different initial CIT concentrations.

	Disinfectant	C_0 (μM) [a]	Simulation Formula	Rate Constant (*M*/*s*)	R^2
Stage 1	NaOCl	0.8	y=0.462x+0.00270	5.35×10^{-12}	0.999
	NaOCl	3.2	y= 0.497x+0.00913	5.75×10^{-12}	0.999
	NaOCl	16	y= 0.481x+0.0310	5.57×10^{-12}	0.999
	NaOCl	32	y=0.661x−0.0296	7.65×10^{-12}	0.999
Stage 2	NaOCl	0.8	y= 0.0460x+0.440	5.32×10^{-13}	0.934
	NaOCl	3.2	y= 0.0403x+0.507	4.66×10^{-13}	0.901
	NaOCl	16	y= 0.0490x+0.495	5.67×10^{-13}	0.900
	NaOCl	32	y=0.0688x+0.556	7.96×10^{-13}	0.956
Stage 1	ClO_2	0.8	y= 0.416x−0.0256	4.81×10^{-12}	0.999
	ClO_2	3.2	y= 0.445x−0.0235	5.15×10^{-12}	0.999
	ClO_2	16	y= 0.410x+0.0261	4.74×10^{-12}	0.999
	ClO_2	32	y=0.303x+0.0538	3.51×10^{-12}	0.999
Stage 2	ClO_2	0.8	y= 0.0405x+0.377	4.69×10^{-13}	0.958
	ClO_2	3.2	y= 0.0354x+0.417	4.09×10^{-13}	0.932
	ClO_2	16	y= 0.0370x+0.434	4.28×10^{-13}	0.908
	ClO_2	32	y=0.0375x+0.354	4.34×10^{-13}	0.900

[a] Initial CIT concentration.

Table 2. Kinetics of NDMA generation during CIT oxidation with NaOCl and ClO_2 at different disinfectant doses.

	Disinfectant	Disinfectant Dose (mM)	Simulation Formula	Rate Constant (*M*/*s*)	R^2
Stage 1	NaOCl	0.1	y=0.178x+0.0108	2.06×10^{-12}	0.999
	NaOCl	0.5	y= 0.269x+0.0470	3.11×10^{-12}	0.998
	NaOCl	1	y= 0.487x+0.0275	5.64×10^{-12}	0.999
	NaOCl	2	y=1.04x+0.0202	1.02×10^{-11}	0.999
Stage 2	NaOCl	0.1	y=0.00798x+0.192	9.23×10^{-14}	0.889
	NaOCl	0.5	y= 0.0254x+0.337	2.94×10^{-13}	0.930
	NaOCl	1	y=0.0541x+0.492	6.26×10^{-13}	0.926
	NaOCl	2	y=0.0869x+1.07	1.01×10^{-12}	0.887
Stage 1	ClO_2	0.1	y= 0.124x+0.0268	1.44×10^{-12}	0.998
	ClO_2	0.5	y= 0.252x+0.0117	2.92×10^{-12}	0.999
	ClO_2	1	y= 0.330x+0.0539	3.82×10^{-12}	0.999
	ClO_2	2	y=0.460x+0.165	5.32×10^{-12}	0.997
Stage 2	ClO_2	0.1	y= 0.00396x+0.189	4.58×10^{-14}	0.772
	ClO_2	0.5	y=0.0206x+0.288	2.38×10^{-13}	0.863
	ClO_2	1	y= 0.0451x+0.359	5.21×10^{-13}	0.908
	ClO_2	2	y=0.0538x+0.709	6.23×10^{-13}	0.820

Table 3. Kinetics of NDMA generation during CIT oxidation with NaOCl and ClO_2 at different pH values.

	Disinfectant	pH	Simulation Formula	Rate Constant (*M*/*s*)	R^2
Stage 1	NaOCl	6	y=0.451x+0.0149	5.21×10^{-12}	0.999
	NaOCl	7	y=0.486x+0.0283	5.62×10^{-12}	0.999
	NaOCl	8	y= 0.462x+0.0283	5.35×10^{-12}	0.999
	NaOCl	9	y=0.502x+0.0256	5.81×10^{-12}	0.999
	NaOCl	10	y=0.454x+0.0202	5.25×10^{-12}	0.998
Stage 2	NaOCl	6	y=0.0436x+0.492	5.06×10^{-13}	0.876
	NaOCl	7	y=0.0541x+0.492	6.26×10^{-13}	0.899
	NaOCl	8	y= 0.0502x+0.489	5.81×10^{-13}	0.893
	NaOCl	9	y=0.0502x+0.510	5.81×10^{-13}	0.919
	NaOCl	10	y=0.0524x+0.443	6.06×10^{-13}	0.920
Stage 1	ClO_2	6	y=0.297x+0.0445	3.44×10^{-12}	0.999
	ClO_2	7	y= 0.330x+0.0539	3.82×10^{-12}	0.998
	ClO_2	8	y=0.303x+0.0526	3.51×10^{-12}	0.999
	ClO_2	9	y=0.351x+0.0701	4.06×10^{-12}	0.999
	ClO_2	10	y=0.294x+0.0633	3.40×10^{-12}	0.999
Stage 2	ClO_2	6	y=0.0370x+0.362	4.28×10^{-13}	0.791
	ClO_2	7	y=0.0446x+0.363	5.16×10^{-13}	0.903
	ClO_2	8	y=0.0358x+0.375	4.14×10^{-13}	0.752
	ClO_2	9	y=0.0305x+0.447	3.53×10^{-13}	0.718
	ClO_2	10	y=0.0373x+0.362	4.31×10^{-13}	0.774

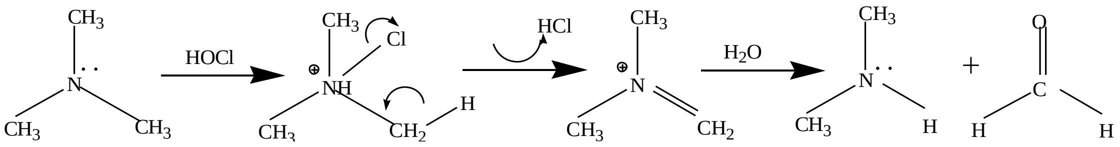

Scheme S1. Mechanism of tertiary alkylamines degradation during chlorination [42].

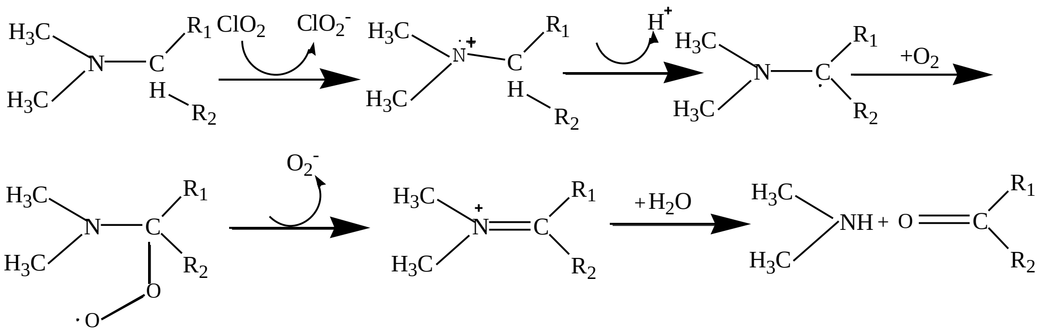

Scheme S2. Mechanism of the reaction of tertiary amines containing NDMA-precursors with ClO_2 [43]
